# The Cost of Opportunity: Anti-Black Discrimination in High Resource Settings

**DOI:** 10.31586/jbls.2024.1128

**Published:** 2024-11-19

**Authors:** Shervin Assari, Hossein Zare

**Affiliations:** 1 Department of Internal Medicine, Charles R. Drew University of Medicine and Science, Los Angeles, CA, United States; 2 Department of Family Medicine, Charles R. Drew University of Medicine and Science, Los Angeles, CA, United States; 3 Department of Urban Public Health, Charles R. Drew University of Medicine and Science, Los Angeles, CA, United States; 4 Marginalization-Related Diminished Returns (MDRs) Center, Los Angeles, CA, United States; 5 Department of Health Policy and Management, Johns Hopkins Bloomberg School of Public Health, Baltimore, MD, United States; 6 School of Business, University of Maryland Global Campus (UMGC), Adelphi, MD, United States

**Keywords:** Racial Disparities, Educational Outcomes, School Discrimination, School Discipline, Childhood Opportunity Index, Academic Achievement, Black-White Achievement Gap

## Abstract

**Objective:**

Inequalities exist in children’s educational outcomes—including reading proficiency, school discrimination, and school disciplinary actions—across zip codes with different levels of educational childhood opportunity index (COI). This study examines the interaction between race and educational environment on children’s educational outcomes. We hypothesize that race, parental education, and their interaction are associated with perceived school discrimination, which in turn reduces their cognitive, academic, and emotional wellbeing. We also hypothesize that Black children with high socioeconomic status (SES) report high perceived school discrimination in high-COI settings.

**Methods:**

Data were drawn from the Adolescent Brain Cognitive Development (ABCD) study, which measures a wide range of educational, cognitive, and emotional outcomes. At the same time, the ABCD children are sampled across areas with vast differences in COI rankings, that can be classified into these five categories: very high, high, average, low, and very low educational COIs. Our structural equation models (SEM) tested the additive and interactive effects of race and educational attainment on perceived school discrimination, and the effects of school discrimination on various cognitive abilities (reading proficiency, picture vocabulary, and list sorting working memory), school suspension, as well as depressed mood. Our multi-group SEM assessed how these relationships vary across educational COI levels.

**Results:**

Our findings showed that high SES Black children report highest school discrimination in residential areas with highest COIs. This is based on the observation that the interaction between race and parental education on experiences of school discrimination were only significant in areas with highest COI. Across residential areas with different COI levels, students who experienced higher school discrimination had higher suspension, worse depression, and worse cognitive performance.

**Conclusion:**

While higher COIs are associated with better academic outcomes, Black-White gaps exist in the role of increased COI through increased racial bias that children perceive. These findings underscore the complexity of educational equity, suggesting that improving COI alone is insufficient for eliminating racial disparities in school experiences. Policies should be in place to reduce school-based discrimination against Black students in high COI settings.

## 1. Introduction

Racial disparities in educational outcomes have persisted across generations in the United States, perpetuating cycles of inequality [1,2]. These gaps hinder the future success of Black students and contribute to broad societal disparities, including economic and health inequities [3,4]. Educational achievement is a primary predictor of socioeconomic mobility, making these disparities particularly consequential [5]. For instance, differences in academic performance led to unequal access to higher education, a critical gateway to economic success and social mobility [6–8]. As a result, Black students disproportionately encounter barriers in college admissions, job opportunities, and long-term economic stability [6–12].

Extensive research across fields—education, economics, psychology, and sociology—has examined the root causes of racial gaps in education [13]. Scholars have identified various structural factors that disadvantage Black children, such as limited access to quality educational resources [14,15]. The historical legacies of Jim Crow laws, segregation, and slavery have left enduring impacts, with Black and White communities often residing in vastly different environments. White neighborhoods are generally more affluent, while Black communities more frequently experience poverty, unemployment, and higher crime rates [16–18]. These environmental disparities create unequal educational opportunities [19], further compounded by systemic issues such as underfunded schools and inadequate educational resources in predominantly Black areas [19–23].

Family background, including parental education, family involvement, and marital status, also influences children’s educational outcomes [24–26], though these factors themselves are shaped by broader environmental inequalities rooted in structural racism [27–33]. While some controversial claims suggest that genetic factors may contribute to these disparities [34], a substantial body of evidence indicates that environmental factors, particularly access to educational opportunities, are the primary drivers of these inequities [35–40].

Research by scholars such as Luthar [41–43] and Assari [44] complicates the narrative by suggesting that highly resourced environments—particularly affluent, predominantly White areas—do not always lead to better outcomes for all groups of children. Luthar’s work shows that high-pressure, competitive environments in affluent areas can negatively impact both minority and White youth [41–43,45–49]. In parallel, Assari’s research suggests that in high-opportunity settings, Black students experience heightened levels of discrimination [50–58], which in turn undermines their academic performance [59–61].

Building on this emerging literature, the current study explores the paradoxical effects of high Childhood Opportunity Index (COI) areas on Black students, particularly those from high socioeconomic status (SES) backgrounds [59–61]. We hypothesize that in residential areas with higher COI, Black students, especially those from higher SES families, are more likely to perceive and experience school-based discrimination. This perceived school discrimination, in turn, is expected to diminish their academic achievements despite the apparent advantages of growing up in a high-opportunity environment. In essence, we extend the framework of “Minorities’ Diminished Returns” [62], which posits that structural racism, segregation, and social stratification reduce the returns of SES for Black children. Specifically, we hypothesize that affluent educational environments may exacerbate biases against Black students, limiting the educational and cognitive benefits they might otherwise gain from their socioeconomic position [63].

## 2. Methods

### 2.1. Setting and Design

This study utilized data from the Adolescent Brain Cognitive Development (ABCD) study, a large-scale, longitudinal dataset designed to explore the factors influencing children’s brain development, cognitive functioning, and educational outcomes. The ABCD study is a national longitudinal study, drawing from a diverse population of children across the United States. It collects comprehensive data on participants’ family socioeconomic status (SES), neighborhood characteristics, academic performance, and neuroimaging data related to brain development. The ABCD data is particularly well-suited for examining how environmental and contextual factors—such as educational opportunity—shape cognitive and educational outcomes across diverse groups.

### 2.2. Measures

#### 2.2.1. Moderator (Strata)

##### Educational Child Opportunity Index (COI):

Educational COIs were measured using residential data that capture local school quality, the availability of educational resources, and access to academic enrichment opportunities in the neighborhood. This measure is based on the COI available in the ABCD dataset, which reflects the broader social and educational context in which children are raised. Higher educational COI values indicate greater access to high-quality educational opportunities. For this analysis, educational COI was treated as a five-level ordinal variable, categorized into very high, high, average, low, and very low educational opportunity.

#### 2.2.2. Predictors

##### Race:

Race was self-reported by the participants or their guardians and was dichotomized into Black and White for the purposes of this study. This dichotomization was necessary to explore specific racial disparities in educational outcomes and school experiences.

##### Parental Education:

Parents were asked: Participants were asked, “What is the highest grade or level of school you have completed or the highest degree you have received?”. The same question was asked for the education of partner/spouse. Responses were 0 = never attended/kindergarten only; 1 = 1st grade; 2 = 2nd grade; 3 = 3rd grade; 4 = 4th grade 4; 5 = 5th grade; 6 = 6th grade 6; 7 = 7th grade 7; 8 = 8th grade; 9 = 9th grade; 10 = 10th grade 10; 11 = 11th grade; 12 = 12th grade; 13 = high school graduate; 14 = GED or equivalent diploma; 15 = some college; 16 = associate degree: occupational; 17 = associate degree: academic program; 18 = Bachelor’s degree (ex. BA); 19 = Master’s degree (ex. MA); 20 = professional school degree (ex. MD); and 21 = Doctoral degree. This variable was transformed into Jaeger education coding [64] that ranges from 31 to 46. A higher score indicated higher educational attainment.

##### Race × Parental Education:

We used multiplicative effect of race (0 vs. 1) and Jaeger education coding (ranges from 31 to 46). The variable was 0 for all White children and Jaeger education coding for Black students.

#### 2.2.3. Mediator

##### Perceived School Discrimination:

Perceived school discrimination was assessed through student self-reports using a series of items designed to capture discriminatory experiences within the school setting. These items included questions about unfair treatment by teachers or peers based on race or ethnicity, as shown in Box 1. Response options were coded as 0 for “No” and 1 for “Yes.” Responses were then summed to create an ordinal measure of perceived school discrimination, ranging from zero to three, with higher scores indicating greater experiences of discrimination in school.

#### 2.2.4. Outcomes

##### List Sorting Working Memory Task:

ABCD has measured working memory using the NIH Toolbox List Sorting Working Memory Task. We used the age-corrected score. The resulting score is a continuous variable, where higher scores indicate better working memory.

##### Picture Vocabulary Task:

ABCD has measured language and cognitive ability (learning) using the NIH Toolbox **Picture Vocabulary Task**. We used the age-corrected score. This task assesses a participant’s ability to name the picture which is shown. The resulting score is a continuous variable, where a higher score indicates better cognitive function, reflecting greater learning and language ability.

##### NIH Toolbox Oral Reading Recognition Test:

Reading ability was assessed using the NIH Toolbox Oral Reading Recognition Test. We used the age-corrected score to account for developmental differences across participants. This test measures a student’s ability to recognize, comprehend, and read written words. Like other cognitive scores, the reading ability score is a continuous variable, with higher scores reflecting greater reading proficiency.

##### School Discipline (Suspension):

School disciplinary actions were measured using a self-reported item that asked students whether they had ever been suspended from school. This variable was binary, with responses coded as 1 for students who had been suspended and 0 for those who had not. This measure captures an important aspect of school disciplinary actions often associated with racial disparities and social justice.

##### Depressive Symptoms (Withdrawal):

Depressive symptoms were measured using the depression and withdrawal subscale from the Child Behavior Checklist (CBC). This subscale provides a continuous measure of depressive symptoms, where higher scores indicate greater levels of depression and withdrawal behaviors. The CBC is widely used in research for assessing emotional and behavioral problems in children and adolescents.

#### 2.2.5. Covariates

Several covariates were included to control for other factors that might influence educational outcomes:

#### Child’s age:

Continuous variable to account for developmental differences.

#### Child’s gender:

Dichotomized as male and female.

#### Parental marital status:

Included to account for family structure, which may influence educational experiences and outcomes. This variable was coded as 1 for married and 0 for not married.

### 2.3. Statistical Analysis

We utilized Structural Equation Modeling (SEM) to examine the effects of race, parental education, and their interaction on perceived school discrimination, and subsequently, how perceived school discrimination influenced educational, cognitive, and emotional outcomes across the five levels of childhood educational opportunity. SEM was chosen for its ability to simultaneously model multiple relationships among variables, making it well-suited for assessing the interaction between race and other factors, such as COI. The primary focus of the analysis was on the coefficient of the interaction between race (coded as Black = 1, White = 0) and parental education, which allowed us to quantify the diminished returns of parental education on perceived school discrimination for Black students compared to their White peers.

We modeled the paths from perceived school discrimination to various outcomes, including educational achievement, cognitive performance, and emotional wellbeing. Additionally, we performed multigroup SEM analyses, where the groups were defined based on the different levels of educational opportunity (COI). This approach enabled us to explore whether the relationship between race, parental education, and school discrimination varied across residential areas s with differing levels of childhood educational opportunity. By conducting these multigroup comparisons, we could determine whether higher educational opportunity levels intensified or mitigated the negative impact of perceived school discrimination on Black students’ outcomes.

### 2.4. Ethical Considerations

The ABCD Study adhered to stringent ethical guidelines and was approved by the institutional review boards (IRBs) of all participating research institutions. Parents or legal guardians provided informed consent for their children’s participation, and the children themselves provided assent. To ensure participant privacy and confidentiality, all data were de-identified throughout the research process. These measures ensured compliance with ethical standards and protected the rights and welfare of all participants.

## 3. Results

Table 1 provides the descriptive statistics for the key study variables. The average age of the children in the sample was 9.0 years (SE = 0.005), with a 95% confidence interval ranging from 9.47 to 9.49. Depression levels, represented as Z-scores, had a mean of −0.014 (SE = 0.010), indicating slightly below-average depression symptoms. The mean level of perceived school discrimination was 1.281 (SE = 0.006), with a confidence interval between 1.268 and 1.293.

In terms of race, 72.9% of the sample identified as White (SE = 0.006), and 27.1% identified as Black (SE = 0.006). When broken down by educational childhood opportunity index (COI) levels, 17.1% of the children lived in the lowest opportunity areas, 11.8% in low opportunity areas, 15.3% in average opportunity areas, 21.7% in high opportunity areas, and 34.1% in the highest opportunity areas.

The sample was fairly balanced by gender, with 47.6% of participants identifying as female (SE = 0.007) and 52.4% as male (SE = 0.007). Regarding the marital status of the household, 34.0% of children lived in an unwed household (SE = 0.006), while 66.0% lived in a married household (SE = 0.006).

Finally, the majority of children had not been suspended from school, with 93.7% reporting no suspension (SE = 0.003), and 6.3% having experienced suspension (SE = 0.003).

The correlation matrix presented in Table 2 shows significant associations among the key study variables. Being Black (Race) was significantly correlated with lower reading scores (r = −0.211, p < 0.05), list sorting working memory (r = −0.23, p < 0.05), and picture Vocabulary (r = −0.33, p < 0.05). Black children were also significantly more likely to experience school suspension (r = 0.215, p < 0.05) and report higher levels of perceived school discrimination (r = 0.167, p < 0.05). Age was not significantly correlated with race (r = −0.008, p > 0.05), but was positively associated with a higher likelihood of school suspension (r = 0.042, p < 0.05). Male children were more likely to face school suspension (r = 0.133, p < 0.05) and perceive higher school discrimination (r = 0.090, p < 0.05). Living in a married household was associated with better cognitive function (r = 0.094, p < 0.05), higher reading scores (r = 0.206, p < 0.05), and lower rates of both perceived school discrimination (r = −0.117, p < 0.05) and school suspension (r = −0.183, p < 0.05). Depression was not significantly associated with race (r = 0.038, p < 0.05), but was positively correlated with school suspension (r = 0.130, p < 0.05) and school discrimination (r = 0.078, p < 0.05).

**Figure 1.**
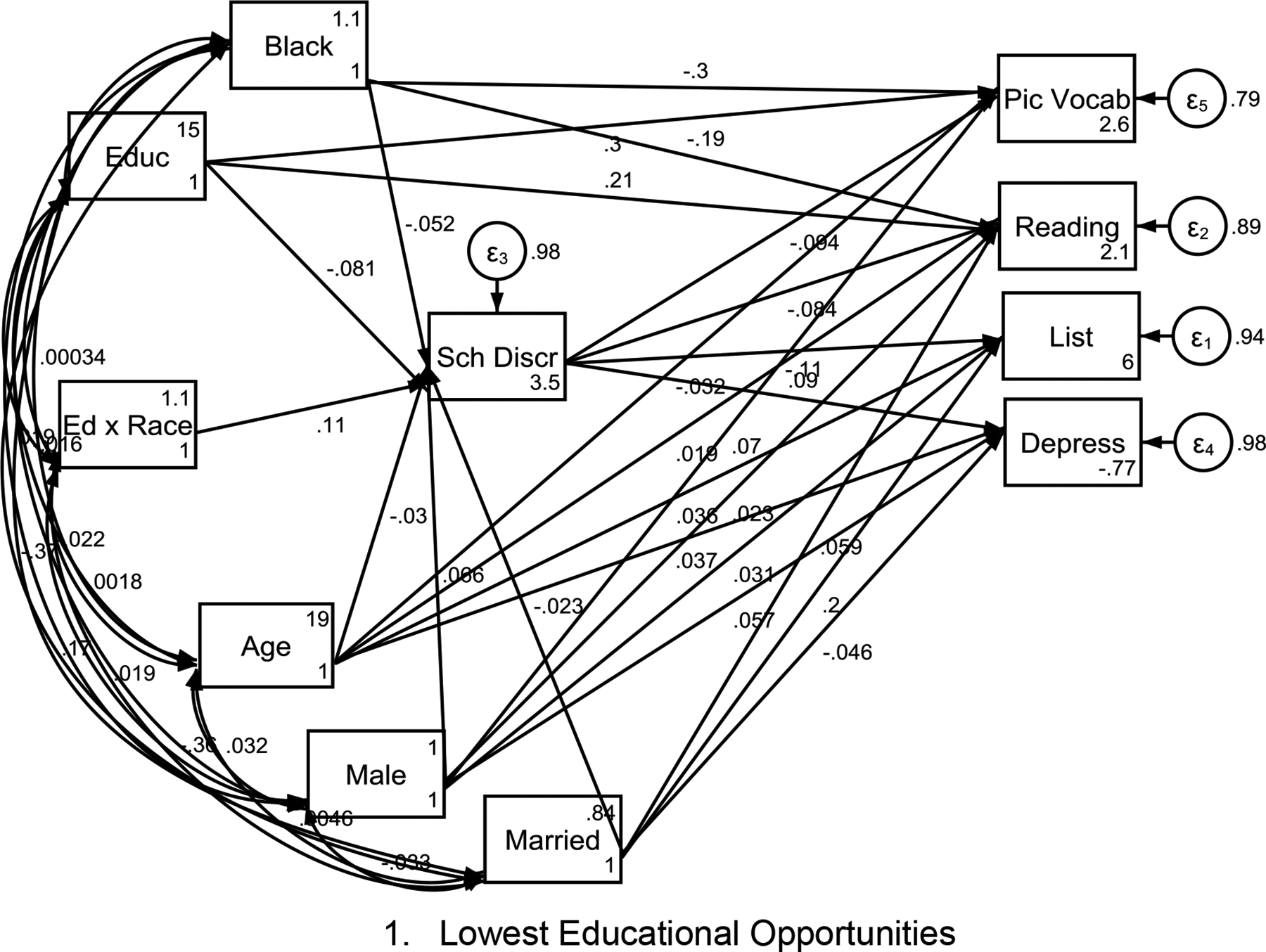

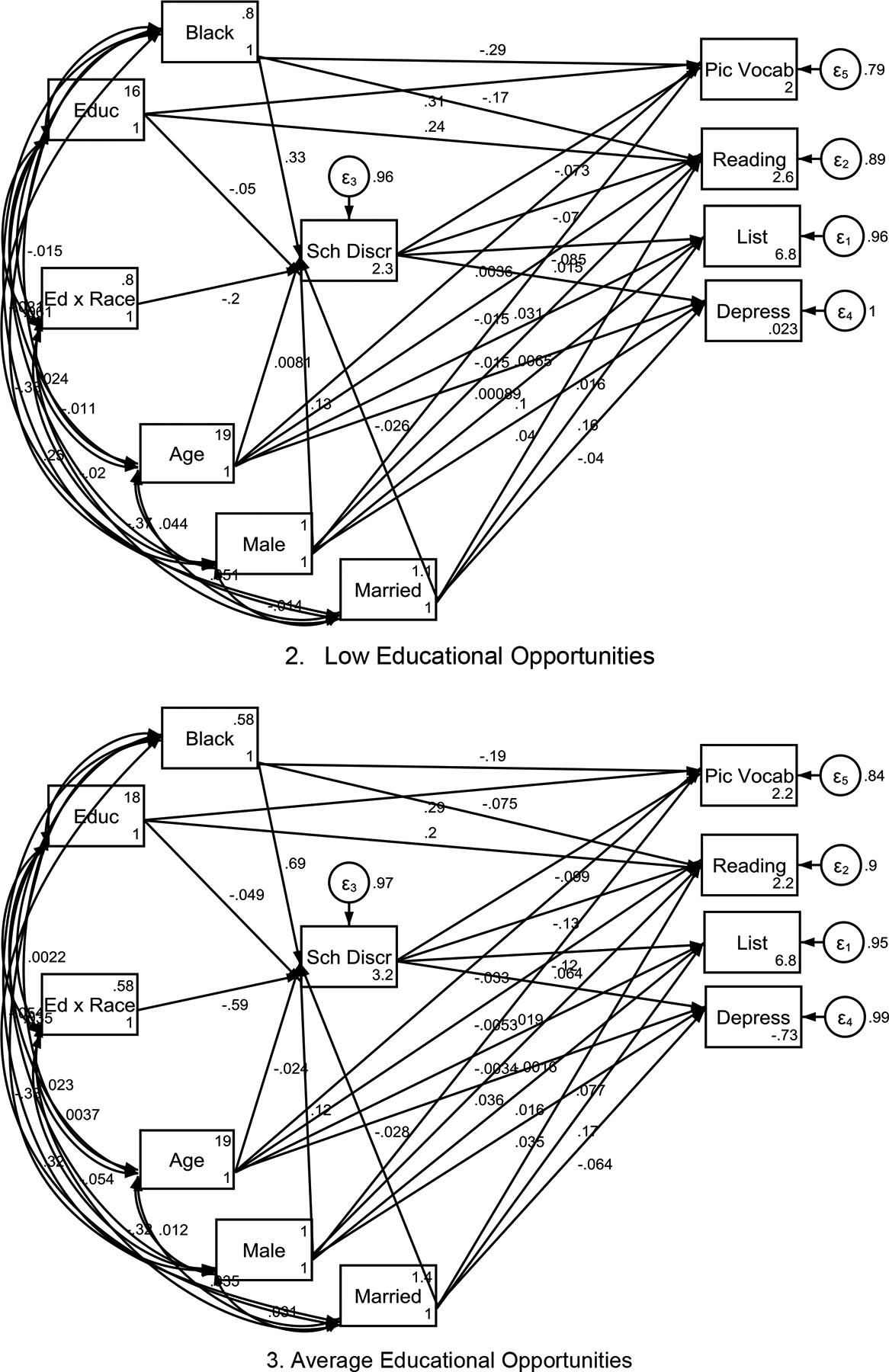

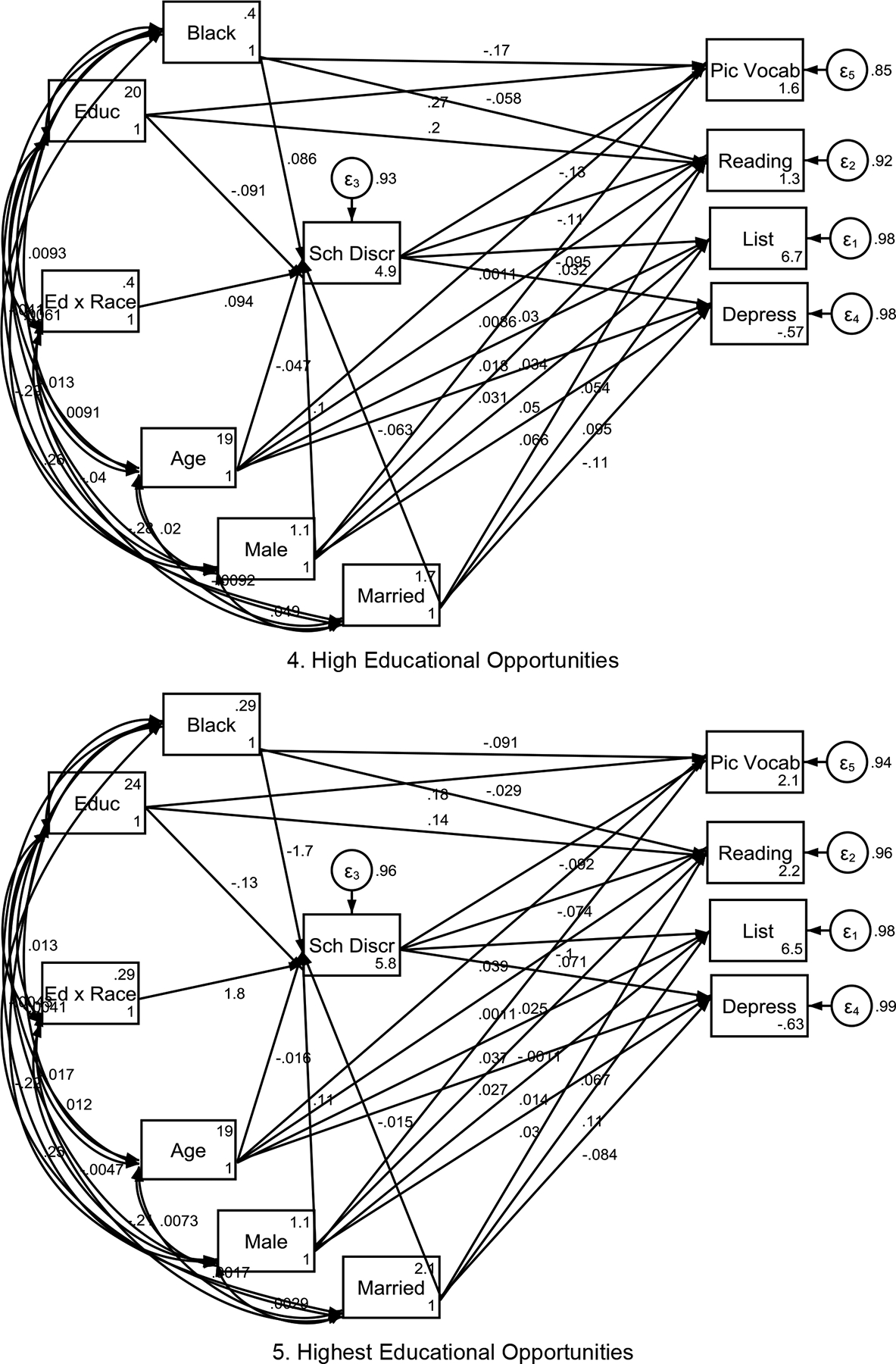
Race by parental education effects on students’ experiences of discrimination and educational, cognitive, and emotional outcomes

Table 3 presents a summary of the multi-group Structural Equation Models (SEMs) across groups defined by different levels of educational childhood opportunity index (COI). Figure 1-1 to 1-5 show the models. Our findings showed that Black-White disparities to the disadvantage of high discrimination of high SES Black children can be seen in areas with highest COIs. This is based on the observation that interaction between race and parental education on experiences of discrimination were only significant in highest-opportunity areas. Then, across areas with different COI levels, Black students experienced a disproportionately higher school discrimination had higher suspension, worse depression, and worse cognitive performance.

## 4. Discussion

This study sets out to examine the effects of race, parental education, and educational opportunity on perceived school discrimination and subsequent educational and emotional outcomes. We hypothesized that while higher levels of educational COI would generally be associated with improved outcomes, Black students—particularly those from high-SES backgrounds—would experience increased discrimination in these high-opportunity environments, thereby limiting the benefits of their SES.

The first key takeaway from our analysis is that higher parental education is associated with lower perceived school discrimination overall. This finding aligns with prior research suggesting that higher SES, reflected through parental education, generally reduces the perception of discrimination among children, youth, and adults. More educated parents tend to have greater social capital, better knowledge of how to navigate institutional systems, and the ability to advocate for their children more effectively. These factors can shield their children from certain forms of discrimination, at least in typical school environments. For the general population, higher parental education and SES are typically protective factors that buffer against the negative effects of discrimination.

However, a more concerning and nuanced finding from our study is that, in areas with the highest COI, high-SES Black students report significantly higher levels of perceived discrimination compared to their White peers. This suggests that in the most advantaged educational settings—where resources, school quality, and opportunities for academic success are highest—Black students continue to face disproportionately high levels of unfair treatment by teachers and school staff. These findings challenge the assumption that improving access to high-quality educational environments will automatically close racial gaps in school experiences and outcomes.

Our results align with the Marginalization-Related Diminished Returns (MDRs) framework, which posits that Black individuals, despite having access to higher SES resources (such as education or income), often experience fewer positive outcomes from these resources due to systemic discrimination and bias. In this context, even though Black students in high-COI areas theoretically have greater opportunities for success, their experiences of heightened racial bias may negate or even reverse the expected benefits of these educational environments. Discrimination can lead to lower academic engagement, reduced sense of belonging, and decreased emotional wellbeing, all of which negatively impact academic outcomes. Thus, rather than benefiting equally from their high SES, Black students may be placed at a unique disadvantage in these high-opportunity environments due to the persistence of racial bias.

The finding that higher COI is associated with increased experiences of school discrimination and suspension rates for Black students highlights an important challenge for policymakers and educators striving for educational equity. It suggests that simply improving the overall quality of educational opportunities is insufficient to address the unique barriers faced by Black students, especially those from higher SES backgrounds. Without tackling the underlying racial biases present in schools, high-COI environments may unintentionally perpetuate or exacerbate racial disparities.

Moreover, our results point to the critical need for systemic interventions that address the roots of school-based discrimination. Anti-racist training for educators, administrators, and school staff is essential for raising awareness of implicit biases and discriminatory behaviors that may disproportionately affect Black students. Reforms in school disciplinary policies, such as adopting restorative justice approaches rather than punitive measures like suspension, are necessary to reduce racial disparities in discipline. Additionally, increasing accountability for discriminatory practices within schools can help create a more equitable environment where all students, regardless of race, can thrive.

These results are also indicative of “Minority Diminished Returns” (MDRs) [65–69]. Diminished returns of family are supported by evidence showing that Black children often do not benefit from socioeconomic resources to the same extent as their White counterparts. Despite achieving similar levels of parental education and income, Black families frequently face structural barriers such as residential segregation, discrimination in housing and employment, and underfunded schools in predominantly Black neighborhoods [27,28,70–75]. These systemic inequalities reduce the impact of family SES on educational opportunities. For example, research has shown that in affluent neighborhoods, Black families encounter discrimination and isolation [50,55,76]. Additionally, systemic discrimination in school systems, including disparities in disciplinary practices and tracking, further limits Black children’s access to the full range of educational opportunities, undermining the potential benefits of parental education and income [77]. These structural factors contribute to the weaker association between family SES and educational outcomes for Black children, highlighting the need for policies that address racial inequities at multiple levels, from housing to school funding.

In addition to diminished educational opportunities, our findings may explain why we observe weaker developmental benefits typically associated with higher SES for Black than White children [78]. This aligns with previous research showing that Black children from high SES backgrounds often exhibit weaker brain development outcomes compared to their White peers [79–83]. Factors such as chronic stress, exposure to trauma, and limited access to mental health resources may contribute to this discrepancy, reducing the impact of parental education and income on Black children’s brain development.

### 4.1. Limitations

Despite the robust dataset from the ABCD study and the advanced modeling techniques used, this research has several limitations. First, the cross-sectional nature of the data limits our ability to establish causal relationships between COI and racial disparities in educational outcomes. Longitudinal data would provide a clearer picture of how these disparities evolve over time and whether changes in educational opportunity or school policies influence the observed outcomes. Second, our reliance on COI rankings as a measure of educational opportunity may oversimplify the complex factors that contribute to school quality and student success. Future research could incorporate more nuanced measures of school environments, including teacher quality, curriculum diversity, and peer support. Lastly, the study does not account for potential variation in individual student experiences within the same areas, which may be influenced by factors such as socioeconomic status, neighborhood segregation, or parental involvement.

### 4.2. Next Research Steps

Future research should aim to build on these findings by addressing the limitations mentioned above. Longitudinal studies that track students over time could offer deeper insights into the causal relationships between educational opportunities, racial disparities, and student outcomes. Additionally, future studies should explore the intersection of race with other variables such as gender, immigration status, and socioeconomic background to understand how different forms of marginalization interact with educational opportunities and bias. Researchers should also examine specific school policies and practices—such as zero-tolerance discipline policies and school-level anti-racism initiatives—that may influence racial disparities in disciplinary actions and discrimination. Finally, future work could investigate interventions designed to reduce school-based discrimination and disciplinary disparities, assessing their effectiveness in reducing the racial gap in both academic achievement and experiences of unfair treatment. By expanding our understanding of these dynamics, future research can contribute to more comprehensive strategies for promoting racial equity in education.

### 4.3. Implications

Our study also has important implications for future research. While we focused on the interaction between race, parental education, and educational opportunity, future work should explore other factors that may influence the experiences of Black students in high-COI residential areas, such as neighborhood racial composition, peer dynamics, and teacher-student relationships. Additionally, longitudinal research is needed to examine how these experiences of discrimination affect long-term outcomes, including college enrollment, career success, and mental health. Addressing these gaps in the literature will provide a more comprehensive understanding of how racial disparities in educational experiences and outcomes can be effectively mitigated.

## 5. Conclusion

In conclusion, this study underscores the complexity of addressing racial disparities in education. While improving educational opportunities is critical, it must be accompanied by targeted efforts to reduce school-based discrimination. Creating equitable educational environments where Black students can fully benefit from their socioeconomic resources requires both structural reforms and a commitment to challenging and dismantling the racial biases that persist within schools.

## Figures and Tables

**Table 1.** Descriptive Data Overall

	Mean	Std. Err.	[95% Conf.	Interval]
Age (Years)	9.0	0.005	9.470	9.490
Picture Vocabulary (Age Corrected)	101.092	0.183	100.734	101.450
List Sorting Working Memory (Age Corrected)	101.229	0.167	100.902	101.556
Reading (Age Corrected)	103.065	0.192	102.688	103.442
Depression (Z score)	−0.014	0.010	−0.033	0.006
Perceived School Discrimination	1.281	0.006	1.268	1.293
	Proportion	SE	[95% conf.	interval]
Race				
White	0.729	0.006	0.717	0.740
Black	0.271	0.006	0.260	0.283
Educational COI				
Lowest	0.171	0.005	0.161	0.182
Low	0.118	0.004	0.110	0.127
Average	0.153	0.005	0.144	0.163
High	0.217	0.006	0.206	0.228
Highest	0.341	0.006	0.328	0.353
Gender				
Female	0.476	0.007	0.463	0.489
Male	0.524	0.007	0.511	0.537
Marital Status of the Household				
Unwed Household	0.340	0.006	0.327	0.352
Married Household	0.660	0.006	0.648	0.673
School Suspension				
No	0.937	0.003	0.931	0.944
Yes	0.063	0.003	0.056	0.069

Educational Childhood Opportunity Index (COI)

**Table 2.** Pearson Correlation between Study Variables

	1	2	3	4	5	6	7	8	9	10	11
1 Race (Black)	1.00										
2 Parental Education	−0.29*	1.00									
3 Married Household	−0.41*	0.36*	1.00								
4 Age	−0.01	0.02	0.02	1.00							
5 Gender (Male)	−0.02	0.00	0.01	0.02	1.00						
6 School Discrimination	0.17*	−0.15*	−0.12*	−0.03*	0.09*	1.00					
7 Picture Vocabulary	−0.33*	0.38*	0.28*	0.01	0.03*	−0.17*	1.00				
8 Reading Ability	−0.21*	0.29*	0.21*	0.01	0.01	−0.14*	0.49*	1.00			
9 List Sorting Working Memory	−0.23*	0.29*	0.21*	0.03*	0.04*	−0.13*	0.38*	0.36*	1.00		
10 Depression	0.04*	−0.11*	−0.10*	0.03*	0.05*	0.08*	−0.01	−0.03*	−0.04*	1.00	
11 School Suspension	0.22*	−0.14*	−0.18*	0.04*	0.13*	0.11*	−0.14*	−0.11*	−0.09*	0.13*	1.00

* p<0.05

**Table 3.** Summary of structural equation model (SEM) across educational COI levels

Independent Variable			Dependent Variable	B	SE	95%	CI	p
**Lowest Educational COI**								
Perceived School Discrimination	→		List Sorting Working Memory	−0.107	0.026	−0.157	−0.057	< 0.001
Age	→		List Sorting Working Memory	0.036	0.024	−0.011	0.084	0.132
Gender (Male)	→		List Sorting Working Memory	0.031	0.024	−0.016	0.078	0.201
Married Household	→		List Sorting Working Memory	0.199	0.024	0.153	0.246	< 0.001
Intercept	→		List Sorting Working Memory	6.031	0.469	5.111	6.951	< 0.001
Perceived School Discrimination	→		Reading Ability	−0.084	0.025	−0.133	−0.036	0.001
Age	→		Reading Ability	0.019	0.023	−0.026	0.065	0.407
Gender (Male)	→		Reading Ability	0.023	0.023	−0.023	0.068	0.335
Married Household	→		Reading Ability	0.059	0.026	0.009	0.110	0.021
Parental Education	→		Reading Ability	0.213	0.023	0.167	0.259	< 0.001
Race (Black)	→		Reading Ability	−0.193	0.027	−0.245	−0.140	< 0.001
Intercept	→		Reading Ability	2.110	0.584	0.966	3.255	< 0.001
Age	→		Perceived School Discrimination	−0.030	0.026	−0.081	0.021	0.247
Gender (Male)	→		Perceived School Discrimination	0.066	0.026	0.015	0.117	0.011
Married Household	→		Perceived School Discrimination	−0.023	0.029	−0.079	0.034	0.428
Parental Education	→		Perceived School Discrimination	−0.081	0.036	−0.151	−0.011	0.024
Parental Education × Race (Black)	→		Perceived School Discrimination	0.106	0.457	−0.790	1.003	0.816
Race (Black)	→		Perceived School Discrimination	−0.052	0.459	−0.951	0.847	0.910
Intercept	→		Perceived School Discrimination	3.501	0.746	2.038	4.964	< 0.001
Perceived School Discrimination	→		Depression	0.090	0.026	0.040	0.140	< 0.001
Age	→		Depression	0.037	0.024	−0.010	0.085	0.124
Gender (Male)	→		Depression	0.057	0.024	0.009	0.104	0.021
Married Household	→		Depression	−0.046	0.024	−0.094	0.002	0.061
Intercept	→		Depression	−0.771	0.456	−1.665	0.124	0.091
Perceived School Discrimination	→		Picture Vocabulary	−0.094	0.024	−0.141	−0.047	< 0.001
Age	→		Picture Vocabulary	−0.032	0.022	−0.076	0.011	0.143
Gender (Male)	→		Picture Vocabulary	0.070	0.022	0.027	0.113	0.002
Parental Education	→		Picture Vocabulary	0.305	0.022	0.262	0.347	< 0.001
Race (Black)	→		Picture Vocabulary	−0.305	0.022	−0.348	−0.261	< 0.001
Intercept	→		Picture Vocabulary	2.560	0.556	1.471	3.649	< 0.001
**Low Educational COI**								
Perceived School Discrimination		→	List Sorting Working Memory	−0.085	0.030	−0.144	−0.027	0.004
Age		→	List Sorting Working Memory	−0.015	0.028	−0.070	0.041	0.604
Gender (Male)		→	List Sorting Working Memory	0.102	0.028	0.047	0.157	< 0.001
Married Household		→	List Sorting Working Memory	0.164	0.028	0.110	0.219	< 0.001
Intercept		→	List Sorting Working Memory	6.832	0.541	5.771	7.893	< 0.001
Perceived School Discrimination		→	Reading Ability	−0.070	0.029	−0.128	−0.012	0.017
Age		→	Reading Ability	−0.015	0.027	−0.068	0.038	0.582
Gender (Male)		→	Reading Ability	0.006	0.027	−0.047	0.060	0.812
Married Household		→	Reading Ability	0.016	0.030	−0.044	0.075	0.607
Parental Education		→	Reading Ability	0.237	0.027	0.184	0.291	< 0.001
Race (Black)		→	Reading Ability	−0.173	0.031	−0.234	−0.112	< 0.001
Intercept		→	Reading Ability	2.576	0.665	1.273	3.880	< 0.001
Age		→	Perceived School Discrimination	0.008	0.029	−0.049	0.066	0.782
Gender (Male)		→	Perceived School Discrimination	0.126	0.029	0.069	0.183	< 0.001
Married Household		→	Perceived School Discrimination	−0.026	0.033	−0.091	0.039	0.437
Parental Education		→	Perceived School Discrimination	−0.050	0.036	−0.120	0.021	0.166
Parental Education × Race (Black)		→	Perceived School Discrimination	−0.202	0.592	−1.364	0.959	0.733
Race (Black)		→	Perceived School Discrimination	0.332	0.596	−0.837	1.500	0.578
Intercept		→	Perceived School Discrimination	2.256	0.785	0.717	3.795	0.004
Perceived School Discrimination		→	Depression	0.015	0.031	−0.045	0.075	0.620
Age		→	Depression	0.001	0.028	−0.055	0.056	0.975
Gender (Male)		→	Depression	0.040	0.028	−0.016	0.096	0.160
Married Household		→	Depression	−0.040	0.028	−0.095	0.016	0.164
Intercept		→	Depression	0.023	0.526	−1.009	1.055	0.965
Perceived School Discrimination		→	Picture Vocabulary	−0.073	0.027	−0.127	−0.020	0.007
Age		→	Picture Vocabulary	0.004	0.026	−0.047	0.054	0.888
Gender (Male)		→	Picture Vocabulary	0.031	0.026	−0.019	0.082	0.225
Parental Education		→	Picture Vocabulary	0.305	0.025	0.256	0.354	< 0.001
Race (Black)		→	Picture Vocabulary	−0.290	0.026	−0.342	−0.238	< 0.001
Intercept		→	Picture Vocabulary	1.975	0.626	0.748	3.202	0.002
**Average Educational COI**								
Perceived School Discrimination		→	List Sorting Working Memory	−0.116	0.025	−0.165	−0.068	< 0.001
Age		→	List Sorting Working Memory	−0.003	0.024	−0.050	0.043	0.886
Gender (Male)		→	List Sorting Working Memory	0.016	0.024	−0.031	0.063	0.505
Married Household		→	List Sorting Working Memory	0.173	0.024	0.127	0.220	< 0.001
Intercept		→	List Sorting Working Memory	6.834	0.467	5.918	7.749	< 0.001
Perceived School Discrimination		→	Reading Ability	−0.128	0.024	−0.175	−0.081	< 0.001
Age		→	Reading Ability	−0.005	0.023	−0.051	0.040	0.820
Gender (Male)		→	Reading Ability	−0.002	0.023	−0.048	0.044	0.946
Married Household		→	Reading Ability	0.077	0.026	0.026	0.127	0.003
Parental Education		→	Reading Ability	0.201	0.024	0.153	0.248	< 0.001
Race (Black)		→	Reading Ability	−0.075	0.026	−0.126	−0.024	0.004
Intercept		→	Reading Ability	2.204	0.619	0.990	3.417	< 0.001
Age		→	Perceived School Discrimination	−0.024	0.025	−0.072	0.025	0.340
Gender (Male)		→	Perceived School Discrimination	0.117	0.025	0.069	0.165	< 0.001
Married Household		→	Perceived School Discrimination	−0.028	0.028	−0.083	0.026	0.310
Parental Education		→	Perceived School Discrimination	−0.049	0.031	−0.109	0.012	0.114
Parental Education × Race (Black)		→	Perceived School Discrimination	−0.588	0.498	−1.564	0.387	0.237
Race (Black)		→	Perceived School Discrimination	0.689	0.502	−0.296	1.673	0.170
Intercept		→	Perceived School Discrimination	3.176	0.707	1.790	4.562	< 0.001
Perceived School Discrimination		→	Depression	0.064	0.025	0.014	0.114	0.012
Age		→	Depression	0.036	0.024	−0.011	0.084	0.133
Gender (Male)		→	Depression	0.035	0.024	−0.012	0.083	0.145
Married Household		→	Depression	−0.064	0.024	−0.111	−0.016	0.009
Intercept		→	Depression	−0.726	0.457	−1.623	0.170	0.112
Perceived School Discrimination		→	Picture Vocabulary	−0.099	0.023	−0.145	−0.054	< 0.001
Age		→	Picture Vocabulary	−0.033	0.022	−0.077	0.011	0.138
Gender (Male)		→	Picture Vocabulary	0.019	0.023	−0.026	0.063	0.409
Parental Education		→	Picture Vocabulary	0.291	0.022	0.247	0.334	< 0.001
Race (Black)		→	Picture Vocabulary	−0.187	0.023	−0.233	−0.141	< 0.001
Intercept		→	Picture Vocabulary	2.163	0.593	1.001	3.325	< 0.001
**High Educational COI**								
Perceived School Discrimination		→	List Sorting Working Memory	−0.095	0.021	−0.137	−0.054	< 0.001
Age		→	List Sorting Working Memory	0.018	0.020	−0.021	0.057	0.356
Gender (Male)		→	List Sorting Working Memory	0.050	0.020	0.011	0.089	0.012
Married Household		→	List Sorting Working Memory	0.095	0.020	0.056	0.134	< 0.001
Intercept		→	List Sorting Working Memory	6.734	0.398	5.954	7.513	< 0.001
Perceived School Discrimination		→	Reading Ability	−0.112	0.020	−0.152	−0.072	< 0.001
Age		→	Reading Ability	0.009	0.019	−0.029	0.046	0.655
Gender (Male)		→	Reading Ability	0.034	0.019	−0.004	0.072	0.083
Married Household		→	Reading Ability	0.054	0.021	0.013	0.094	0.009
Parental Education		→	Reading Ability	0.201	0.020	0.162	0.240	< 0.001
Race (Black)		→	Reading Ability	−0.058	0.021	−0.100	−0.017	0.006
Intercept		→	Reading Ability	1.297	0.558	0.204	2.390	0.020
Age		→	Perceived School Discrimination	−0.047	0.020	−0.086	−0.008	0.017
Gender (Male)		→	Perceived School Discrimination	0.102	0.020	0.063	0.141	< 0.001
Married Household		→	Perceived School Discrimination	−0.063	0.022	−0.105	−0.020	0.004
Parental Education		→	Perceived School Discrimination	−0.091	0.023	−0.135	−0.046	< 0.001
Parental Education × Race (Black)		→	Perceived School Discrimination	0.094	0.420	−0.730	0.917	0.824
Race (Black)		→	Perceived School Discrimination	0.086	0.423	−0.743	0.914	0.839
Intercept		→	Perceived School Discrimination	4.945	0.587	3.794	6.095	< 0.001
Perceived School Discrimination		→	Depression	0.032	0.021	−0.009	0.073	0.131
Age		→	Depression	0.031	0.020	−0.008	0.070	0.121
Gender (Male)		→	Depression	0.066	0.020	0.027	0.105	0.001
Married Household		→	Depression	−0.106	0.020	−0.145	−0.067	< 0.001
Intercept		→	Depression	−0.572	0.382	−1.321	0.177	0.134
Perceived School Discrimination		→	Picture Vocabulary	−0.127	0.020	−0.166	−0.089	< 0.001
Age		→	Picture Vocabulary	0.001	0.019	−0.035	0.038	0.951
Gender (Male)		→	Picture Vocabulary	0.030	0.019	−0.007	0.067	0.108
Parental Education		→	Picture Vocabulary	0.267	0.018	0.231	0.303	< 0.001
Race (Black)		→	Picture Vocabulary	−0.172	0.020	−0.210	−0.133	< 0.001
Intercept		→	Picture Vocabulary	1.561	0.537	0.508	2.614	0.004
**Highest Educational COI**								
Perceived School Discrimination		→	List Sorting Working Memory	−0.104	0.017	−0.138	−0.071	< 0.001
Age		→	List Sorting Working Memory	0.037	0.016	0.004	0.069	0.027
Gender (Male)		→	List Sorting Working Memory	0.014	0.017	−0.018	0.047	0.383
Married Household		→	List Sorting Working Memory	0.106	0.016	0.074	0.138	< 0.001
Intercept		→	List Sorting Working Memory	6.544	0.328	5.903	7.186	< 0.001
Perceived School Discrimination		→	Reading Ability	−0.074	0.017	−0.107	−0.041	< 0.001
Age		→	Reading Ability	0.001	0.016	−0.031	0.033	0.945
Gender (Male)		→	Reading Ability	−0.001	0.016	−0.033	0.031	0.945
Married Household		→	Reading Ability	0.067	0.017	0.033	0.101	< 0.001
Parental Education		→	Reading Ability	0.143	0.017	0.110	0.176	< 0.001
Race (Black)		→	Reading Ability	−0.029	0.018	−0.063	0.006	0.106
Intercept		→	Reading Ability	2.208	0.521	1.188	3.229	< 0.001
Age		→	Perceived School Discrimination	−0.016	0.017	−0.049	0.016	0.324
Gender (Male)		→	Perceived School Discrimination	0.113	0.017	0.080	0.145	< 0.001
Married Household		→	Perceived School Discrimination	−0.015	0.018	−0.050	0.020	0.400
Parental Education		→	Perceived School Discrimination	−0.126	0.019	−0.162	−0.089	< 0.001
Parental Education × Race (Black)		→	Perceived School Discrimination	1.763	0.370	1.037	2.489	< 0.001
Race (Black)		→	Perceived School Discrimination	−1.678	0.372	−2.407	−0.949	< 0.001
Intercept		→	Perceived School Discrimination	5.777	0.542	4.714	6.840	< 0.001
Perceived School Discrimination		→	Depression	0.071	0.017	0.037	0.104	< 0.001
Age		→	Depression	0.027	0.016	−0.005	0.059	0.098
Gender (Male)		→	Depression	0.030	0.017	−0.003	0.062	0.072
Married Household		→	Depression	−0.084	0.016	−0.116	−0.052	< 0.001
Intercept		→	Depression	−0.631	0.313	−1.246	−0.017	0.044
Perceived School Discrimination		→	Picture Vocabulary	−0.092	0.017	−0.125	−0.059	< 0.001
Age		→	Picture Vocabulary	0.039	0.016	0.008	0.071	0.015
Gender (Male)		→	Picture Vocabulary	0.025	0.016	−0.007	0.057	0.124
Parental Education		→	Picture Vocabulary	0.176	0.016	0.145	0.208	< 0.001
Race (Black)		→	Picture Vocabulary	−0.091	0.017	−0.124	−0.058	< 0.001
Intercept		→	Picture Vocabulary	2.103	0.514	1.096	3.111	< 0.001

Educational Childhood Opportunity Index (COI)

## Box 1. Items of perceived school discrimination

**Table:**

In the past 12 months, have you felt discriminated against: because of your race, ethnicity, or color? Definition of ethnicity: groups of people who have the same customs, or origin How often do the following people treat you unfairly or negatively because of your ethnic background? Teachers: How often do the following people treat you unfairly or negatively because of your ethnic background? Other students
